# Modification of TiO_2_ nanotubes by graphene–strontium and cobalt molybdate perovskite for efficient hydrogen evolution reaction in acidic medium

**DOI:** 10.1038/s41598-022-27143-5

**Published:** 2022-12-30

**Authors:** Mariusz Szkoda, Anna Ilnicka, Malgorzata Skorupska, Marcin Wysokowski, Jerzy P. Lukaszewicz

**Affiliations:** 1 Faculty of Chemistry, Department of Chemistry and Technology of Functional Materials, Gdańsk University of Technology, Narutowicza 11/12, 80-233 Gdańsk, Poland; 2 Advanced Materials Center, Gdańsk University of Technology, Narutowicza 11/12, 80-233 Gdańsk, Poland; 3 Faculty of Chemistry, Nicolaus Copernicus University in Torun, Gagarina 7, 87-100 Torun, Poland; 4 Faculty of Chemical Technology, Institute of Chemical Technology and Engineering, Poznan University of Technology, Berdychowo 4, 60-965 Poznań, Poland; 5 Centre for Modern Interdisciplinary Technologies, Nicolaus Copernicus University in Torun, Wilenska 4, 87-100 Torun, Poland

**Keywords:** Energy science and technology, Materials science

## Abstract

Herein, we demonstrate that modification of TiO_2_ nanotubes with graphene–strontium and cobalt molybdate perovskite can turn them into active electrocatalysts for hydrogen evolution reaction (HER). For this purpose, a simple method of hydrothermal synthesis of perovskites was developed directly on the TiO_2_ nanotubes substrate. Moreover, the obtained hybrids were also decorated with graphene oxide (GO) during one-step hydrothermal synthesis. The obtained materials were characterized by scanning electron microscopy with energy dispersive X-ray analysis, Raman spectroscopy, and X-ray diffraction analysis. Catalytic properties were verified by electrochemical methods (linear voltammetry, chronopotentiometry). The obtained hybrids were characterized by much better catalytic properties towards hydrogen evolution reaction compared to TiO_2_ and slightly worse than platinum. The optimized hybrid catalyst (decorated by GO) can drive a cathodic current density of 10 mA cm^−2^ at an overpotential of 121 mV for HER with a small Tafel slope of 90 mV dec^−1^ in 0.2 M H_2_SO_4_.

## Introduction

Hydrogen evolution reaction (HER) is a crucial reaction in water splitting. It is still required to synthesize inexpensive HER electrocatalysts in a way that is effective, straightforward, and ecologically benign. The design of the appropriate core–shell structure of the catalyst, and controlling thereof shape affect the activity and durability of the catalyst^1^. A three-dimensional structure of the catalyst is beneficial for exposing the surface area, and furnishing the active sites, as well as is propitious to the diffusion and adsorption of hydrogen molecules, expediting the HER process^2, 3^. Furthermore, the three-dimensional structure of the catalyst influences oxygen evolution reaction performance^4^. A core–shell hierarchical nanostructure of a catalyst composed of many conductive interconnected networks, provides multiple channels for ionic or electron delivery^5^. The plasma-treated sponge-like nanoalloy exhibits high HER performance due to the exposure of more active and edge sites^6^. Besides the structure of the catalyst, another factor affecting the properties of the catalyst is its doping with, for example, nitrogen heteroatoms^7^. Compare to the well-known and expensive platinum catalyst the TiO_2_ is widely explored as an alternative catalyst. However, to enhance the electrocatalytic activity of TiO_2_ different modifications are needed. In some papers, TiO_2_ has been coupled with metals like nickel^8^, ruthenium^9, 10^, gold^11^, cobalt^12^, metal oxides like Co_3_O_4_^13^, BiVO_4_^14^, or metal–organic frameworks^15^. Very promising materials designed with TiO_2_ to catalyze the HER are metal sulfides, e.g., MoS_2_^16–19^_,_ CoS_2_^20^, or WS_2_^21^. Also, hybrid composites with polymers like truxene-based porous organic polymer^22^ or poly(aniline)^23^, poly(o-phenylenediamine), poly(thiophene), or poly(pyrrole)^24^ have proved to be desired candidates to enhance photocatalytic activity of TiO_2_. Another way to modify the properties of electrocatalyst is doping with non-metals. As described in many papers sulfur-doping, nitrogen-doping, or carbon-doping reduce bandgap of TiO_2_^25–27^. Another way to improve properties was described by Pandey et al. where in theoretical predictions a hybrid structure of the ionic liquid 1-ethyl-3-methylimidazolium trifluoromethanesulfonate and (TiO_2_)_n_ nanoclusters (with n = 2−12) has been investigated in the pursuit of new catalyst materials for effective HER^28^. Furthermore, hybrids of TiO_2_ and carbon materials like graphene oxide^13, 29^ and multi-wall carbon nanotubes^30^. Nanocomposites of graphene oxide with metal–organic framework described in the literature, were also efficient electrocatalyst for hydrogen production via Volmer and Heyrovsky mechanisms^31^. The disadvantage of many solutions is still doping with expensive metals like silver, platinum^29^. Therefore, it is important to find new solutions but without expensive metals and easy to preparation.

In this paper, a novel hybrid materials of CoMoO_4_, SrMoO_4_, or their composition SrMoO_4_ and CoMoO_4_ deposited on TiO_2_ nanotubes surface and supported with graphene oxide (GO) were achieved by hydrothermal method. Here, uniform TiO_2_ nanotubes were used as support to growth CoMoO_4_ or SrMoO_4_. The morphology was investigated by scanning electron microscopy (SEM) with energy dispersive X-ray analysis (EDX), Raman spectroscopy, and X-ray diffraction analysis (XRD). These hybrids were subjected as active catalysts for hydrogen evolution reaction in acidic medium. Compared with pristine TiO_2_ and commercial Pt, the optimal hybrid could attain a current density 10 mA cm^−2^ at a low overpotential of 121 mV with a Tafel slope of 90 mV dec^−1^. In addition, the hybrid displayed better cycling stability and durability.

## Results and discussions

### Physicochemical characterization

**Figure 1:**
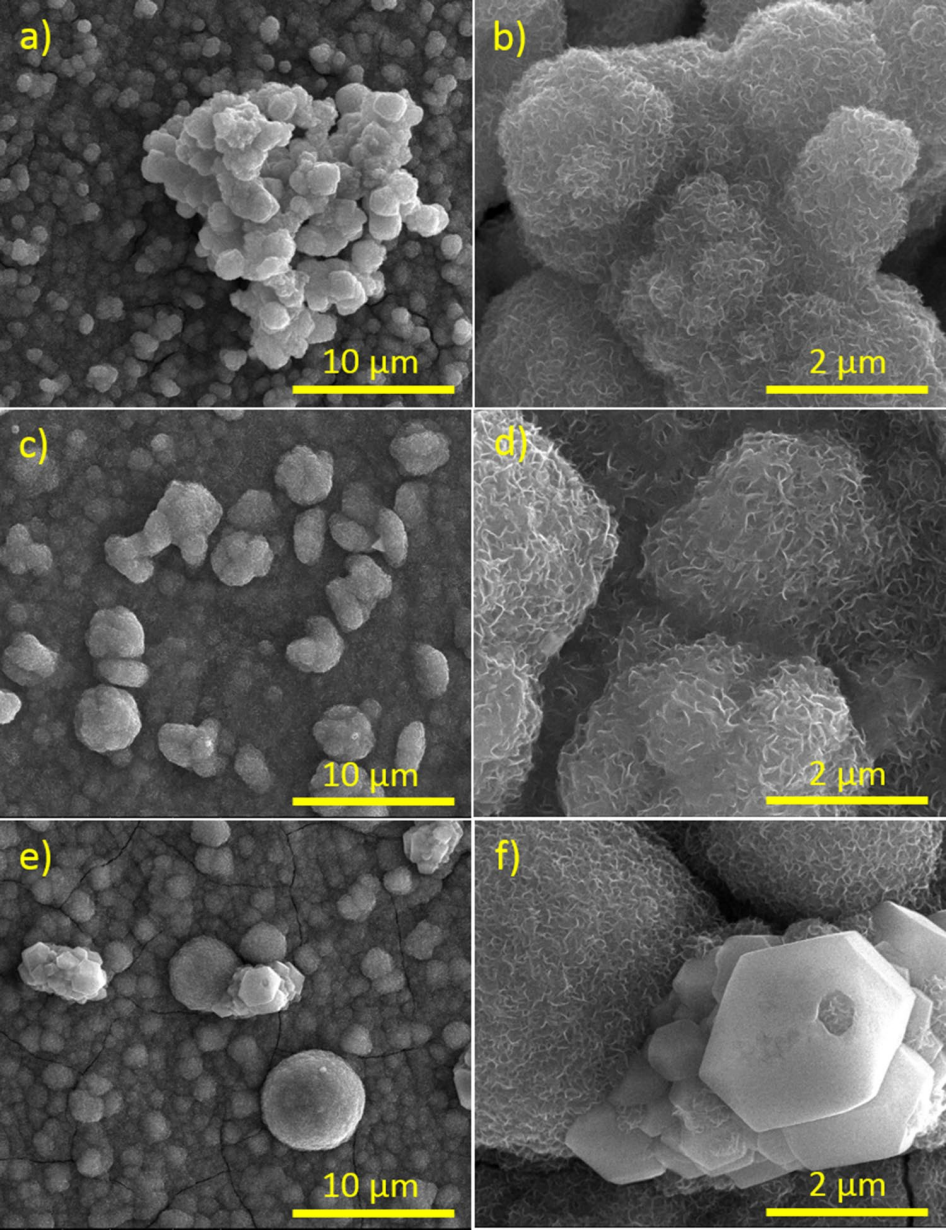
SEM images of samples: (**a,b**) TiO_2_/CoMoO_4_, (**c,d**) TiO_2_/SrMoO_4_, (**e,f**) TiO_2_/SrMoO_4_/CoMoO_4_.

The morphology of the as-prepared samples was characterized by scanning electron microscopy. As shown in Fig. 1a, on the surface of sample TiO_2_/CoMoO_4_ the TiO_2_ nanotubes are not visible (pure TiO_2_ nanotubes are presented in Figure S1) because during the hydrothermal process the CoMoO_4_ particles densely covered TiO_2_ surface. Intriguingly, each CoMoO_4_ microspheres (Fig. 1b) is actually a three dimensionally interconnected porous structure and is assembled from numerous nanospheres. For TiO_2_/CoMoO_4_ sample the diameter of CoMoO_4_ particles ranges from 1 to 2 µm. It can be observed that the particles agglomerate. The surface of TiO_2_/SrMoO_4_ sample (Fig. 1c) clearly demonstrated that the product considered of a large amount of spherical structures covered TiO_2_ surface and shows the similar morphology to TiO_2_/CoMoO_4_ sample. The diameters of SrMoO_4_ spheres are about 2 µm. A higher-magnification SEM image (Fig. 1d) reveals that an individual sphere is composed of tens of similar nanosheets. These nanosheets are connected with each other to form a sphere with random orientation. Figure 1e,f illustrate the SEM images for TiO_2_/SrMoO_4_/CoMoO_4_ sample with different magnifications. Figure 1f shows the high magnification from which it can be seen that particles deposited on the TiO_2_ surface do not possess a uniform size and shape. The mean diameters of three-dimensional microspheres are greater and fluctuate between 2 and 4 µm. These nanosheets are connected with each other to form nanosheets-based microstructures with random orientation. Furthermore, the surface of some of the crystals is very smooth. In addition, the energy dispersive X-ray analysis also confirms the SrMoO_4_ and CoMoO_4_ structure (Fig. S2).

**Figure 2:**
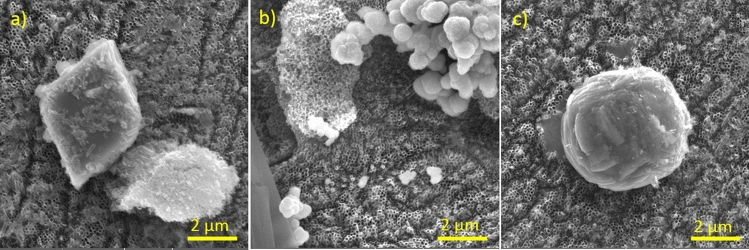
SEM images of samples: (**a**) TiO_2_/SrMoO_4_/GO, (**b**) TiO_2_/CoMoO_4_/GO, (**c**) TiO_2_/SrMoO_4_/CoMoO_4_/GO.

The morphologies and microstructures of the hybrid materials include of TiO_2,_ SrMoO_4_ CoMoO_4,_ and GO are present in Fig. 2. These images confirm that applying hydrothermal technology can prepare in one sample three-dimensional structures with different morphology. Figure 2a for TiO_2_/SrMoO_4_/GO sample indicates the SrMoO_4_ are randomly distributed on the TiO_2_ surface. Figure 2b displays that the as-prepared sample TiO_2_/CoMoO_4_ has microstructure with CoMoO_4_ agglomerated on the surface. It can also be found that these microspheres are consisted of a large number of nanosheets.

The surface morphology of CoMoO_4_ with GO is comparable to sample without GO. The agglomeration of particles impacts on their size distribution and the average diameter is 1 µm. For sample TiO_2_/SrMoO_4_/CoMoO_4_/GO (Fig. 2c) addition of GO caused to stronger agglomeration of SrMoO_4_ and CoMoO_4_ to spherical structures. To visualize the distribution of Co, Mo, Sr, Ti, O, S, and C, energy dispersive X-ray spectroscopy mapping was performed (Fig. S3).

**Figure 3:**
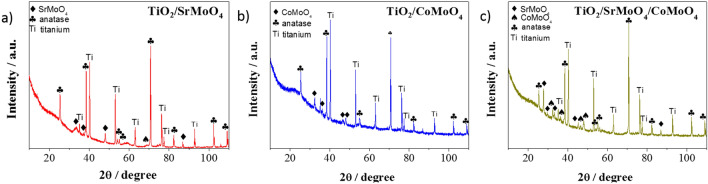
X-ray diffraction spectra of samples: (**a**) TiO_2_/SrMoO_4_, (**b**) TiO_2_/CoMoO_4_, (**c**) TiO_2_/SrMoO_4_/CoMoO_4_.

**Figure 4:**
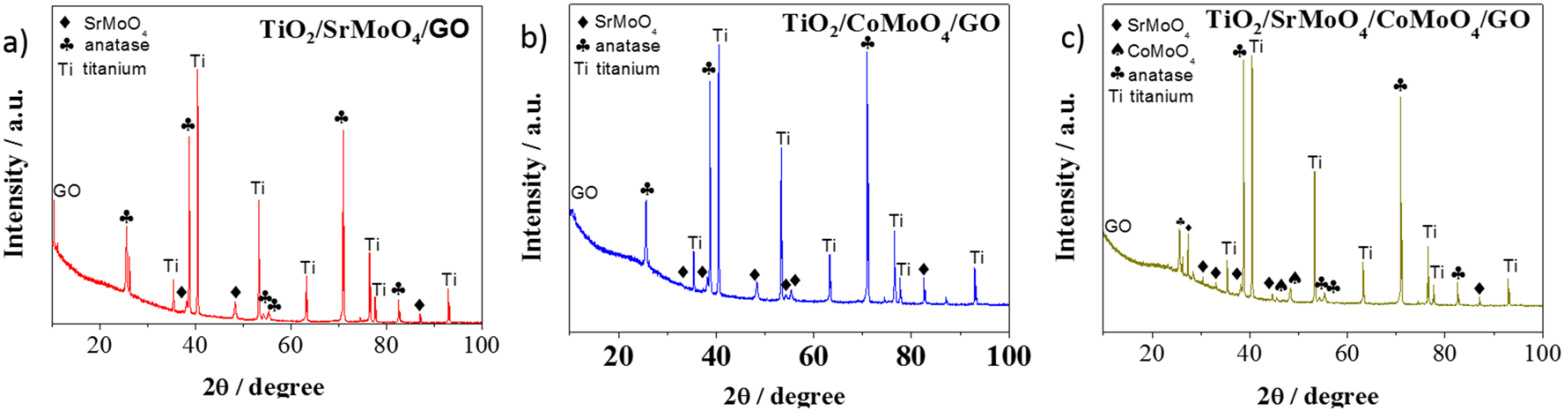
X-ray diffraction spectra of samples: (**a**) TiO_2_/SrMoO_4_/GO, (**b**) TiO_2_/CoMoO_4_/GO, (**c**) TiO_2_/SrMoO_4_/CoMoO_4_/GO.

The obtained materials were also structurally characterized by XRD. Figures 3 and 4 shows the XRD pattern of TiO_2_/SrMoO_4_, TiO_2_/CoMoO_4_, TiO_2_/SrMoO_4_/CoMoO_4_, TiO_2_/SrMoO_4_/GO, TiO_2_/CoMoO_4_/GO, TiO_2_/SrMoO_4_/CoMoO_4_/GO. Some of the SrMoO_4_ and CoMoO_4_ peaks overlap with the intense anatase peaks. However, several characteristic peaks from these compounds can be observed, as indicated by the corresponding symbols in Figs. 3 and 4. However, the low intensity of these peaks proves the low crystallinity of SrMoO_4_ and CoMoO_4_. Nonetheless, the signature peaks of SrMoO_4_ at 33.7, 37.1, 38.5, 48.1, 76.3, 77.4, 82.3, 86.8^32–34^ and of CoMoO_4_ at 25.4, 32.4, 36.3, 38.5, 40.2, 45.2, 63.0^35–37^ are very prominent in all samples^38, 39^. The remaining crystallite phases were indexed as characteristic peaks of anatase and the titanium (Ti) phase acting as TiO_2_ NT support^40^. The ornamental GO structure peak was observed at 10.5 plane for only a TiO_2_/SrMoO_4_/GO hybrid. However, the presence of GO can be better characterized by Raman spectroscopy.

**Figure 5:**
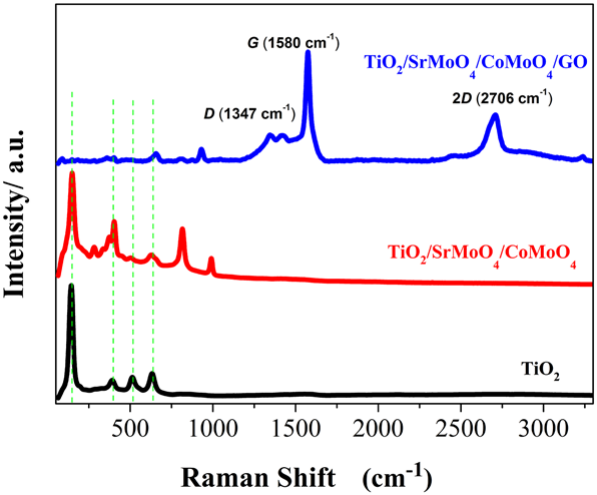
Raman spectra of TiO_2_ nanotubes, TiO_2_/SrMoO_4_/CoMoO_4_, and TiO_2_/SrMoO_4_/CoMoO_4_/GO.

Raman spectra of TiO_2_ nanotubes, TiO_2_/SrMoO_4_/CoMoO_4,_ and TiO_2_/SrMoO_4_/CoMoO_4_/GO within the frequency range 100–3300 cm^−1^ are shown in Fig. 5. Several bands characteristic for the pure anatase crystalline form were identified in samples of pristine TiO_2_ and the hybrid materials. However, in the case of a material with added carbon, the anatase peaks are not that pronounced. The bands located at 143, 398, 516, and 640 cm^−1^ are attributed to E_g_(1), B_1g_, A_1g_, and E_g_(3) active anatase modes, respectively^41, 42^. The band at around 326 cm^−1^ is attributed to the symmetric stretching of the Co–O–Mo bond^43^. The peak of around to 300 cm^−1^ corresponds to Sr–O–Mo bond^44^. The band located at 802 cm^−1^ is associated with asymmetric stretching modes of O–Mo–O bond while the band at 904 cm^−1^ corresponds to the symmetric stretching mode of Mo–O bond^45^. On analyzing the Raman bands of modified material by GO, two distinct bands, namely, D and G bands, were obtained at 1347 cm^−1^ and 1580 cm^−1^. The D band is corresponding to disorder carbon while the G band is attributed to sp^2^ hybridized carbon^46, 47^. This confirmed that the graphene component is maintained during the hydrothermal process. Moreover, the second order of zone boundary phonons or 2D band which is related to the stacking nature of graphene layers was observed at 2706 cm^−1^ for GO^48^.

### Electrocatalytic activities for hydrogen evolution

**Figure 6:**
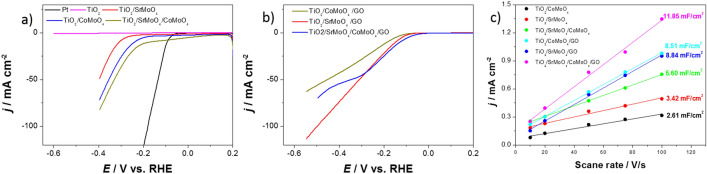
LSV curves of obtained catalysts (**a**) without and (**b**) with the addition of carbon. (**c**) Calculated double-layer capacitance (C_DL_) used to estimate the electrochemically active surface area of the obtained catalysts.

The catalytic activity of obtained hybrids for hydrogen evolution reaction (HER) was measured in 0.2 M H_2_SO_4_ using three-electrode configuration with a scan rate of 5 mV s^−1^. For comparison, bare TiO_2_ nanotubes and Pt were also tested. Figure 6a,b give the HER polarization curves of commercial Pt disc, pure TiO_2_ nanotubes, and the obtained hybrids without and with the addition of carbon.

According to linear sweep voltammograms (LSVs) in Fig. 6a,b, the overpotential values for all electrodes at j_HER_ = 10 mA cm^−2^ are summarized in Table 1. The obtained values indicate that a small amount of carbon has a large share in the HER performance of the obtained hybrids. It is worth noting that the η value of TiO_2_/SrMoO_4_/CoMoO_4_/GO is only 29 mV larger than that of pure Pt and comparable to or smaller than those of various transition metal-based electrocatalyst (Table S1).

**Table 1:** The HER parameters, Tafel slopes and estimated electrochemically active surface area for the obtained catalysts.

Electrode	Overpotential (mV vs. RHE) to achieve a current density of −10 mA cm^−2^	Onset potential (mV vs. RHE)	b (mV dec^−1^)	Estimated ECSA (cm^2^) (0.04 mF cm^−2^)
Pt	92	−75	38	–
TiO_2_	Does not achieve	–	330	–
TiO_2_/SrMoO_4_	313	−275	152	85.5
TiO_2_/CoMoO_4_	242	−219	168	65.25
TiO_2_/SrMoO_4_/CoMoO_4_	167	−209	91	140
TiO_2_/SrMoO_4_/GO	146	−96	62	221
TiO_2_/CoMoO_4_/GO	184	−121	75	212.75
TiO_2_/SrMoO_4_/CoMoO_4_/GO	121	−60	90	296.25

The electrochemically active surface area (ECSA) was determined to elucidate the effect of composition on HER. The ECSA was estimated using the CV method's double-layer capacitance. For example, Fig. S4 shows the CV for TiO_2_/SrMoO_4_/CoMoO_4_ that was performed in the non-Faradaic region at different scanning rates from 0.35 to 0.45 V vs. Ag/AgCl/3 M KCl. For the all obtained electrodes, the charging current at the different scan rates was plotted (Fig. 6c) to obtain the double-layer capacitance from the slope. Can be observed a difference in the double-layer capacitance (C_DL_) between the catalysts depending on the composition. The catalysts modified with graphene oxide have higher C_DL_ values. The ECSA was estimated using the commonly used specific capacity of 0.04 mF cm^−2^^49^. Results are summarized in Table 1. The electrochemically active surface area is higher for materials with added carbon, which may impact better catalytic properties toward hydrogen evolution.

Figure S5 shows the electrochemical impedance spectra recorded for obtained catalysts at an open circuit potential. A slightly increasing slope of the curves, in the case of materials modified by carbon, indicates an improving capacitive behavior. Moreover, the small diameter or the absence of a semicircle, in the case of a material with the addition of graphene oxide, indicates the small charge transfer resistance (Rct)^50^. The Rct is associated with the electrocatalytic kinetics at the catalyst/electrolyte interface and gives information about the reaction rate of HER^51^. In summary, the lower the charge transfer resistance, the better the HER kinetics.

**Figure 7:**
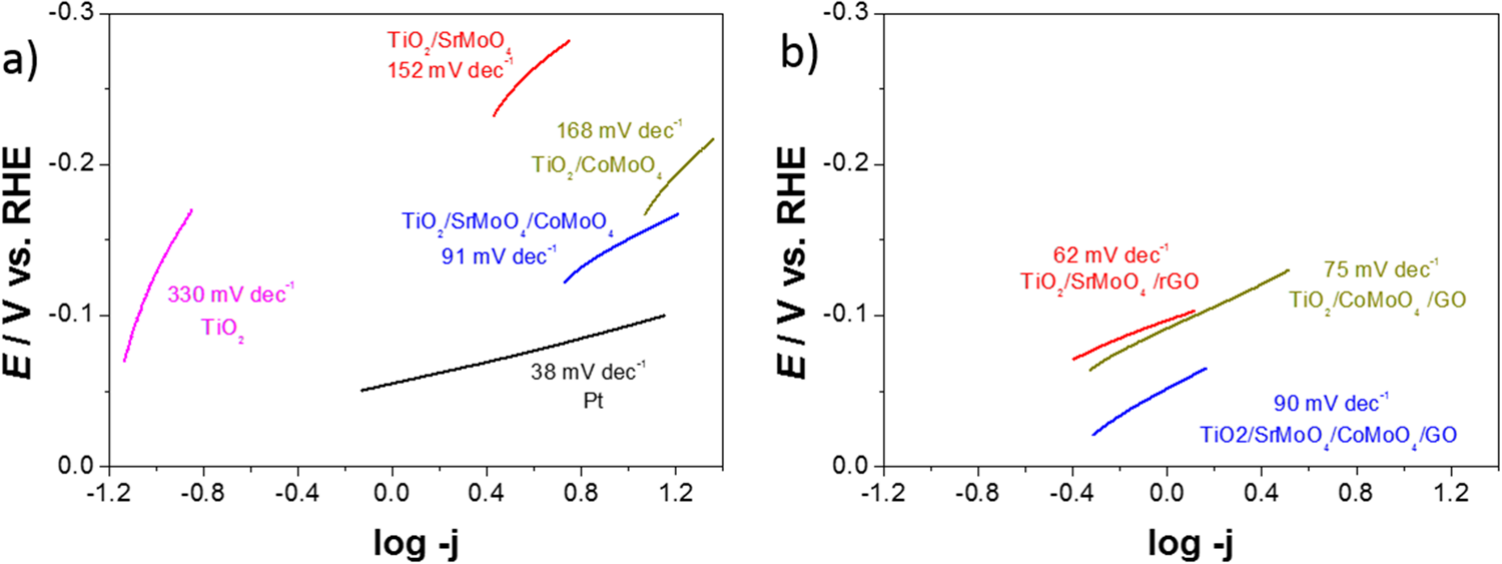
The Tafel plots calculated from the corresponding LSV plots for obtained catalysts (**a**) without and (**b**) with the addition of carbon.

Figure 7 presents the Tafel plots. At high overpotentials the HER on the electrodes is kinetically controlled, which can be given by the Tafel equation^52^:

$$\eta =a+blog\,j$$

where η (V) means overpotential, *a* (V) is the cathodic intercept related to the exchange current density, *b* (V dec^−1^) means the cathodic Tafel slope, and *j* (A cm^−2^) means catalytic current density. Tafel slope values calculated from the linear portion of potential vs. logarithmic value of current density deliver useful kinetic metrics of the catalyst^38^. The Tafel slope values were 38, 330, 152, 168, 91, 62, 75, and 90 for Pt, TiO_2_ nanotubes, TiO_2_/SrMoO_4_, TiO_2_/CoMoO_4_, TiO_2_/SrMoO_4_/CoMoO_4_, TiO_2_/SrMoO_4_/GO, TiO_2_/CoMoO_4_/GO, TiO_2_/SrMoO_4_/CoMoO_4_/GO, respectively. Concerning as-synthesized electrocatalysts, TiO_2_/SrMoO_4_/GO showed the lowest Tafel slope value. In this case, the positive effect of the presence of carbon in all hybrids was also observed. The data of Tafel plots along are also tabulated in Table 1.

**Figure 8:**
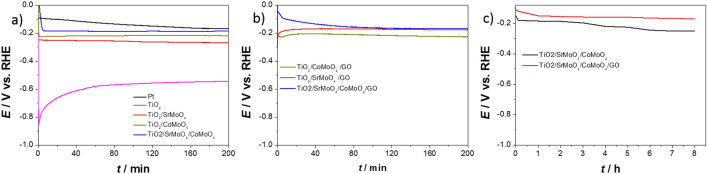
Part variations of overvoltage value at a constant current density of 10 mA cm^−2^ with the processing time for (**a**) without and (**b**) with the addition of carbon. (**c**) Chronopotentiometry measurement with the current density of 10 mA cm^−2^ for 8 h for TiO_2_/SrMoO_4_/CoMoO_4_ and TiO_2_/SrMoO_4_/CoMoO_4_/GO.

To examine the long-term stability of obtained electrodes, chronopotentiometric tests were also performed at the fixed current density of 10 mA cm^−2^ (Fig. 8a,b). As can be seen, almost all electrodes show good stability of overpotential with a polarization current of -10 mA cm^−2^. However, in the case of pure TiO_2_ nanotubes the overpotential had shifted from 842 to 541 mV by the end of 200 min. In other cases, the overpotential oscillates between 261 and 140 mV.

In order to verify the validity of the modification with graphene oxide, an even longer stability test (8 h) was performed for the best materials without and with the addition of carbon. As shown in Fig. 8c, the overpotential of TiO_2_/SrMoO_4_/CoMoO_4_ decreased from −152 to −251 mV during 8 h, indicating lower stability compared with the same material but with the addition of carbon. The results demonstrate that adding GO during synthesis can greatly enhance its stability and electrocatalytic performance for hydrogen evolution in an acidic solution. According to the literature^53–55^, in carbon–metal hybrid materials, transition metals exhibit remarkable catalytic abilities, while the carbon material provides better conductivity, stability and greater surface area. In addition, interactions between carbon and metallic materials can change the properties of the whole hybrid, contributing to the formation of new sites with increased catalytic activity.

## Materials and methods

### TiO_2_ nanotubes preparation

TiO_2_ nanotubes were prepared by one-step anodization of titanium film according to a previously optimized procedure^56^. First, the titanium sheets, placed in a mixture of acetone and isopropanol (1:1 ratio), were cleaned using ultrasound for 20 min. Anodization was carried out in a two-electrode system in which both the anode and cathode were titanium sheets. Electrodes were placed in 50 cm^3^ water:glycol mixture (volume ratio 1:19) about 2 cm separated from each other. The electrolyte contained 0.27 M ammonium fluoride and 1 M phosphoric acid (V). The anodization process was carried out for 2 h at a constant voltage of 40 V. The nanotubes obtained on the titanium foil were washed with a solution of 100 μl HF in 50 cm^3^ water (to remove inorganic impurities), followed by distilled water. In the last step, the obtained material was calcined at 450 °C for 2 h. As a result, TiO_2_ NT were transformed from amorphous to crystalline form.

### Synthesis of hybrids

**Figure 9:**
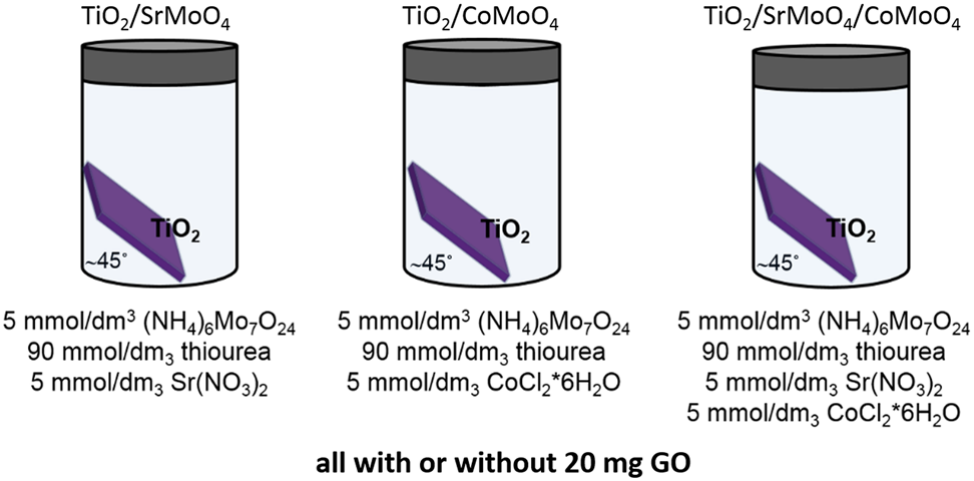
Reagents used in the hydrothermal method and their concentration.

The layer of SrMnO_4_, CoMoO_4_ and SrMoO_4_/CoMoO_4_, respectively, was deposited on TiO_2_ nanotubes by hydrothermal method. Appropriate amounts of each component of the reaction mixture (Fig. 9) were dissolved in 15 mL of deionized water to form a clear solution. Hybrids were made with or without 20 mg of GO added to the reaction mixture.

After that, the as-prepared fresh solution and TiO_2_ nanotubes plate were transferred into a Teflon-lined stainless autoclave (30 mL), which was then tightly sealed and left in an oven at 220 °C for 7 h. After the autoclave cooled down to room temperature, the TiO_2_ nanotube plate with black layer was taken out and washed with deionized water several times to remove unstable products, which was then dried in a vacuum oven at 60 °C.

### Solid state physics techniques

A MIRA3 scanning electron microscope (SEM) (Tescan, Czech Republic) was used to assess the surface. The crystalline phases were characterized by X-ray diffractometer (Philips X”Pert with detector X’Celerator Scientific). Raman spectra were recorded with a Raman spectrometer (Senterra, Bruker Optik) with a green laser (532 nm) used as the excitation source.

### Electrochemical characterization

A three-electrode cell consisting of a working electrode, Pt as a counter electrode and Ag/AgCl (in 3 M KCl) as a reference electrode has been employed. 0.2 M H_2_SO_4_ solution has been used as electrolyte. All the electrochemical measurements have been performed using potentiostat/galvanostat (BioLogic VSP 2078) at room temperature. Unless otherwise specified, all potentials measured were referenced to the reversible hydrogen electrode (RHE) using the following equation: E (RHE) = E (Ag/AgCl) + 0.21 + 0.059 pH. Linear sweep voltammograms were obtained with a scan rate of 1 mV s^−1^. Tafel plots were derived from the corresponding LSV curves. The long-term stability was tested by a chronopotentiometric technique at a −10 mA cm^−2^ current density. The electrochemically active surface area (ECSA) was estimated through the cyclic voltammetry method. Under a non-Faradaic region, a series of CV scans were performed at different scan rates (10, 20, 50, 75, 100 mV s^−1^). The double-layer capacitance (C_DL_) was found by obtaining the slope of the linear fit. The ECSA of obtained electrodes was determined on the basis of the equation: ECSA = $\frac{{C}_{DL}}{{C}_{s}}$, where Cs is the specific capacitance. The C_s_ used for our calculations was 0.040 mF cm^−2^—value commonly used for metals^49, 57^. Moreover, EIS was performed for two of the best materials in a frequency range between 20 kHz and 1 Hz with a voltage amplitude of 10 mV at an open circuit potential.

## Conclusions

An efficient hybrid catalyst in the form of TiO_2_ nanotubes with CoMoO_4_, SrMoO_4_, and graphene oxide on their surface was proposed in this research. The strategy of creating active centers by modification of TiO_2_ with carbon and perovskite-type metal oxide in one synthesis step may pave the way toward the synthesis of cheap, efficient, and stable catalysts. As a result, TiO_2_/SrMoO_4_/CoMoO_4_/GO hybrid was proved to be an efficient and durable HER electrocatalyst in acidic medium. The optimized catalyst only required a low overpotential of 120 mV at 10 mA cm^−2^ with a Tafel slope of 90 mV dec^−1^, superior to results of pristine TiO_2_. The high HER activity and excellent durability of TiO_2_/SrMoO_4_/CoMoO_4_/GO hybrid make it a promising alternative to commercial Pt-based HER catalyst.

## Supplementary Information

Supplementary Information.

## Data Availability

The datasets generated and/or analysed during the current study are available in the BRIDGE OF KNOWLEDGE repository (https://mostwiedzy.pl/en/open-research-data/x-ray-diffraction-spectra-of-modification-of-tio2-nanotubes-by-graphene-strontium-and-cobalt-molybda,1128104009119681-0).
