# Supplementary material for: Modification of TiO2 nanotubes by graphene–strontium and cobalt molybdate perovskite for efficient hydrogen evolution reaction in acidic medium
Source: Sci Rep. 2022 Dec 30;12:22577. doi: 10.1038/s41598-022-27143-5 (PMC9803638; doi:10.1038/s41598-022-27143-5)
Supplement: Supplementary file 1 — Supplementary Information. [file 41598_2022_27143_MOESM1_ESM.docx]

**Modification of TiO_2_ nanotubes by graphene - strontium and cobalt molybdate perovskite for efficient hydrogen evolution reaction in acidic medium**

Mariusz Szkoda ^1,2,*^, Anna Ilnicka ^3^, Malgorzata Skorupska ^3^, Marcin Wysokowski ^4^, and Jerzy P. Lukaszewicz ^3,5^

^1^ Faculty of Chemistry, Department of Chemistry and Technology of Functional Materials, Gdańsk University of Technology, Narutowicza 11/12, 80-233 Gdańsk, Poland

^2^ Advanced Materials Center, Gdańsk University of Technology, Narutowicza 11/12, 80-233 Gdańsk, Poland

^3^ Faculty of Chemistry, Nicolaus Copernicus University in Torun, Gagarina 7, 87-100 Torun, Poland

^4^ Faculty of Chemical Technology, Institute of Chemical Technology and Engineering, Poznan University of Technology, Berdychowo 4, 60-965 Poznań, Poland

^5^ Centre for Modern Interdisciplinary Technologies, Nicolaus Copernicus University in Torun, Wilenska 4, 87-100 Torun, Poland

***** Corresponding author. E-mail address: mariusz.szkoda1@pg.edu.pl


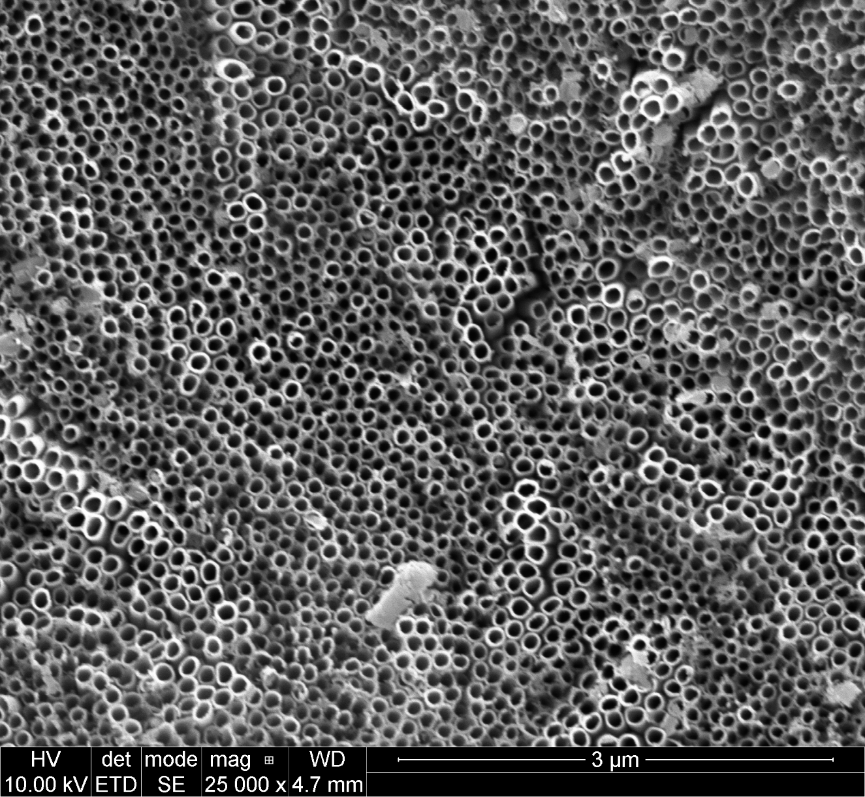


**Figure S1.** SEM image of pure TiO_2_ nanotubes.


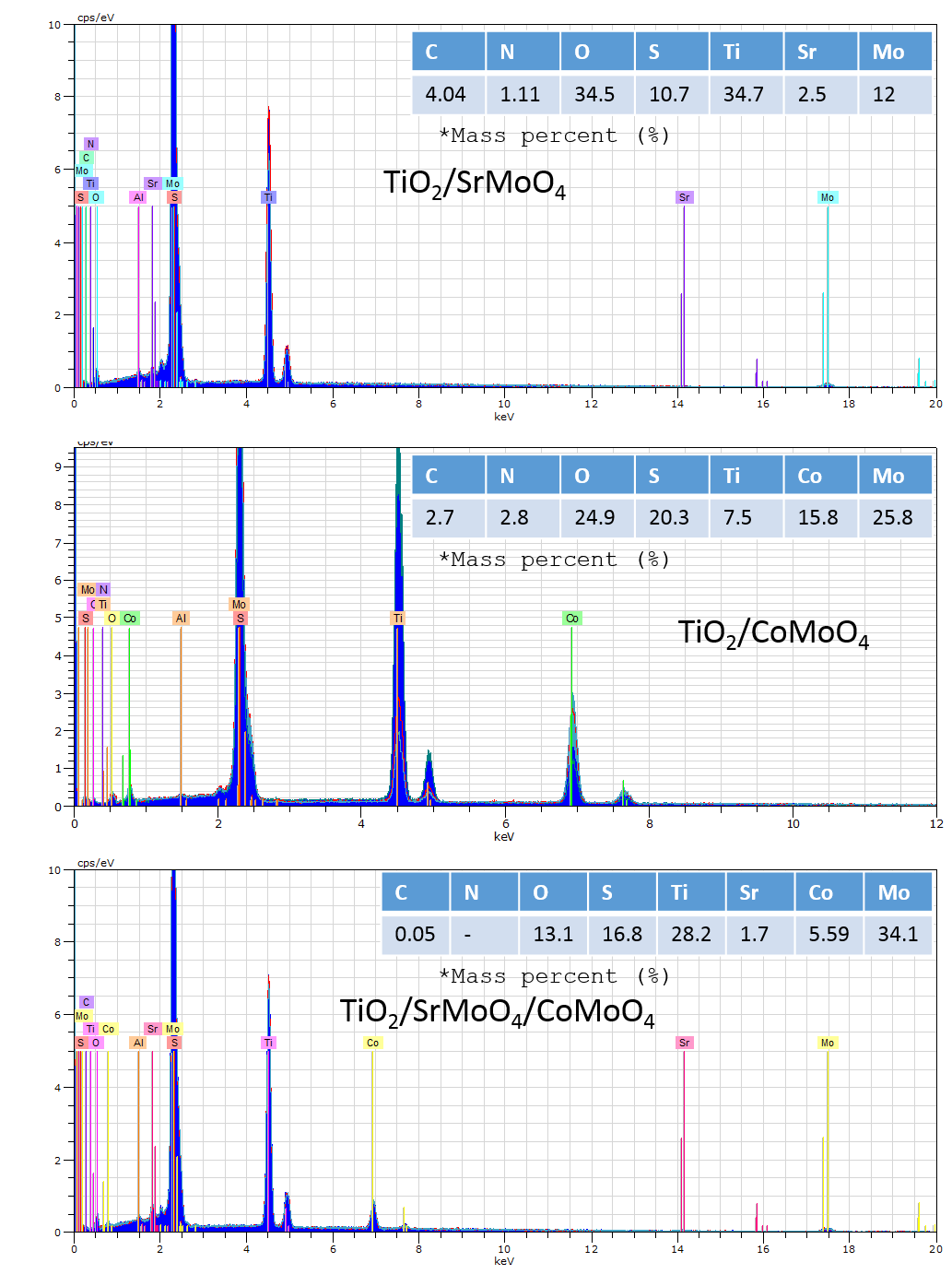


**Figure S2.** EDX spectra of the TiO_2_/SrMoO_4_, TiO_2_/CoMoO_4_, TiO_2_/SrMoO_4_/CoMoO_4_.


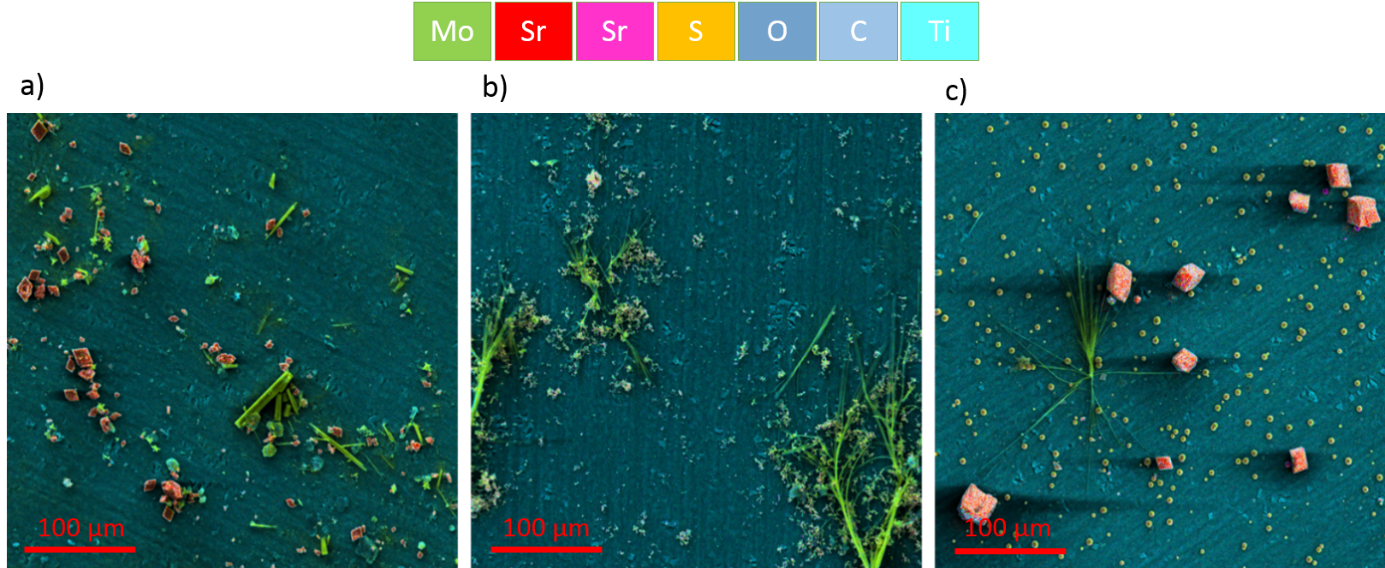


**Figure S3.** EDX elemental distribution mapping of the (a) TiO_2_/SrMoO_4_/GO, (b) TiO_2_/CoMoO_4_/GO, (c) TiO_2_/SrMoO_4_/CoMoO_4_/GO.





**Figure S4.** Cyclic voltammograms of TiO_2_/SrMoO_4_/CoMoO_4_ used to determine the electrochemical double-layer capacitance (Cdl) and estimate the electrochemically active surface area (ECSA). CV were performed on between -0.35 V and -0.45 V vs Ag/AgCl/3M KCl at different scan rates (10, 20 50 75, 100 mV s^−1^).





**Figure S5.** EIS spectra of obtained catalysts.

**Table S1.** Overpotential and Tafel slope of various transition metal-based electrocatalyst described in the literature.

| **Electrocatalyst** | **Overpotential (mV) for *j*_HER_ = 10 mA cm^−2^** | **Tafel slope (mV dec^−1^)** | **Reference** |
| --- | --- | --- | --- |
| SrMoO_4_ | ~800 | 159 | [1] |
| SrMoO_4_@rGO-MWCNT | 197 | 81 | [2] |
| MoS_2_-ZnO | 239 | 62 | [3] |
| Co-CoO/ZnFe_2_O_4_ | 226 | 138 | [4] |
| MoS_2_/α-MoO_3_ | 232 | 81 | [5] |
| CoSe_2_/MoSe_2_ | 90 | 87 | [6] |
| NiCoP/rGO | 124 | 91 | [7] |
| CoOx@CN | 280 |  | [8] |
| Co/CoP | 138 | 72 | [9] |

**References:**

1 Aruna, K.K., Manoharan, R. Electrochemical hydrogen evolution catalyzed by SrMoO_4_ spindle particles in acid water, *Int. J. Hydrogen Energy*. **38,** 12695–12703 (2013).

2 Kareem, A., Kunhiraman, A.K., Maiyalagan, T. Hydrogen evolution reaction catalyzed by microstructured SrMoO_4_ decorated on three-dimensional nanostructured rGO/f-MWCNT in acidic medium, *Ionics (Kiel)*. **26**, 5055–5064 (2020).

3 Sumesh, C.K. Zinc oxide functionalized molybdenum disulfide heterostructures as efficient electrocatalysts for hydrogen evolution reaction, *Int. J. Hydrogen Energy*. **45**, 619–628 (2020).

4 Liu, W., Zhou, Y., Bao, J., Wang, J., Zhang, Y., Sheng, X., Xue, Y., Guo, C., Chen, X. Co-CoO/ZnFe_2_O_4_ encapsulated in carbon nanowires derived from MOFs as electrocatalysts for hydrogen evolution, *J. Colloid Interface Sci*. **561**, 620–628 (2020).

5 Manikandan, A., Ilango, P.R., Chen, C., Wang, Y., Shih, Y., Lee, L., Wang, Z.M., Ko, H., Chueh Y. A superior dye adsorbent towards the hydrogen evolution reaction combining active sites and phase-engineering of (1T/2H) MoS_2_/α-MoO_3_ hybrid heterostructured nanoflowers, *J. Mater. Chem. A.* **6** 15320–15329 (2018)..

6 Xia, L., Song, H., Li, X., Zhang, X., Gao, B., Zheng, Y., Huo, K., Chu, P.K. Hierarchical 0D−2D Co/Mo Selenides as Superior Bifunctional Electrocatalysts for Overall Water Splitting, *Front. Chem.* **8,** 382-392 (2020).

7 Li, J., Yan, M., Zhou, X., Huang, Z., Xia, Z. Mechanistic Insights on Ternary Ni_2_ − xCoxP for Hydrogen Evolution and Their Hybrids with Graphene as Highly Efficient and Robust Catalysts for Overall Water Splitting, *Adv. Funct. Mater.* **26**, 6785–6796 (2016).

8 Jin, H., Wang, J., Su, D., Wei, Z., Pang, Z., Wang, Y. In situ Cobalt–Cobalt Oxide/N-Doped Carbon Hybrids As Superior Bifunctional Electrocatalysts for Hydrogen and Oxygen Evolution, *J. Am. Chem. Soc*. **137**, 2688–2694 (2015).

9 Xue, Z., H. Su, Q. Yu, B. Zhang, H. Wang, X. Li, J. Chen, Janus Co/CoP Nanoparticles as Efficient Mott – Schottky Electrocatalysts for Overall Water Splitting in Wide pH Range, *Adv. Energy Mater*. **7**, 1602355 (2017).
